# The American Dental Society of Europe

**Published:** 1890-04

**Authors:** 


### THE AMERICAN DENTAL SOCIETY OF EUROPE.—SEVENTEENTH ANNUAL MEETING, PARIS, AUGUST 6 AND 7, 1889.

*First Day's Proceedings.—Morning Session.*

The chair was taken, at ten o'clock, by Dr. W. St. George Elliott, president.

#### REPORT OF THE MEMBERSHIP COMMITTEE.

*Dr. Elliott.*—Gentlemen, I want to apologize for the delay this morning. I came here at 9.30, but as I left the minutes of the last meeting at my boarding-house, which is near the Arc de Triomphe, I was obliged to return there and get them. We will commence the meeting by calling the roll, when Dr. Patton, the secretary, will read the minutes of the last meeting, which he proposes to do in a condensed form. I should like to have your opinion on the matter, if that proceeding will be agreeable to you? Our last secretary took the minutes in detail, but he had not apparently the time nor the inclination to write them out afterwards.

It was agreed that the minutes should be read in a condensed form as suggested.

The call of the roll.

*Members Present.*—Dr. L. C. Bryan, Basel; Dr. J. W. Crane, Paris; Dr. I. B. Davenport, Paris; Dr. C. V. Du Bouchet, Paris; Dr. W. St. Geo. Elliott, London; Dr. F. Forster, Berlin; Dr. N. S. Jenkins, Dresden; Dr. W. D. Miller, Berlin; Dr. W. R. Patton, Cologne; Dr. Ed. Rosenthal, Brussels; Dr. W. Sachs, Breslau; Dr. Hoffmann, Wiesbaden; Dr. Schaffner, Florence; Dr. I. H. Spaulding, Paris; Dr. A. Wetzel, Basel; Dr. N. W. Williams, Geneva; Dr. Chas. Adams, Leipsic; Dr. Fred. Merrill, Milan.

*Guests Present.*—Dr. Bonwill, delegate of Odontological Society of Pennsylvania; Dr. Warrington Evans, Washington; Dr. Rosenthal, Sr., Liége, Belgium; Dr. Thomas, Vilbon, Spain; Dr. W. E. Royce, Tunbridge Wells, England; Dr. Mitchell, London; Dr. A. C. Hugenschmidt, Paris; Dr. C. Agabey, Athens, Greece; Dr. F. Du Bouchet, Paris; Dr. Theo. Frick, Paris; Dr. G. C. Daboel, Paris; Dr. C. P. Terry, Milan; Dr. L. A. Obrian, Jr.; Paris; Dr. W. F. Kelsey, Marseilles; Dr. E. A. Bogue, New York; Dr. F. C. Collett, New York.

Dr. Elliott then greeted the guests, thanking them for their presence, etc.

The guests then retired, while the members attended to the official duties of the society. On conclusion of the same, Dr. Elliott opened the professional duties of the meeting.

*Dr. Elliott.*—Dr. Miller has some preparations with him, and it is very desirable that we should have them at once. I would also say that Dr. Bonwill is here, and he and Dr. Mitchell have proposed giving clinics. Dr. Du Bouchet has been kind enough to offer facilities for doing so. Most of us, of course, are interested in the exhibition; but still there may be some members who may perhaps be sufficiently interested to stay for the clinics. Dr. Mitchell proposes to put on a crown, and Dr. Bonwill, particularly, wishes to speak about his articulator and to exhibit his instruments.

*Dr. Miller.*—I think he has already been invited by the Executive Committee.

*Dr. Crane.*—I spoke to him personally about it, as being the only member here.

*Dr. Elliott.*—He wished to give a clinic in London, but I rather dissuaded him from it, as I knew there would not be sufficient general interest to get an audience.

*Dr. Miller.*—He gave us a clinic in Berlin, and I think we had one hundred persons present, mostly students.

*Dr. Elliott.*—I will ask Dr. Miller to give us his communication on the antiseptic action of filling-materials.

*Dr. Miller.*—Gentlemen, I wish to demonstrate some culture experiments, showing the antiseptic action of different filling-materials. It will scarcely be questioned by any one that, in a great many cases, if not in all, the probability of success in filling teeth would be greatly heightened if the filling-material could be made to exert a permanent antiseptic action upon the walls and margin of the cavity. I have tested a great number of different filling-materials, in order to ascertain whether they exert any antiseptic effect; and I have found one material which possesses this action in a high degree,—that is, copper amalgam.

I have employed two different methods for testing the antiseptic action of filling-materials. By the first method I inoculate a tube of ordinary nutritive gelatine with a fungus which grows rapidly at room temperature without melting the gelatine. I liquefy the gelatine and pour it upon a horizontal glass plate. While it is still soft, I drop into it pieces of the filling-material whose antiseptic action is to be tested. A plate so prepared usually becomes cloudy in about twenty-four hours through the development of innumerable colonies of bacteria. If, however, the material possesses an antiseptic action, no fungi will develop in its vicinity, and it will consequently appear surrounded by a zone of transparent gelatine.

In this manner I have tested nearly all the filling-materials now in use. I have found that copper amalgam possesses considerable antiseptic action. Not only freshly mixed amalgam, but even very old fillings and pieces of dentine from teeth which had been filled with copper amalgam, show strong antiseptic action. Gold amalgam possesses very little, and in most cases no action whatever. Phosphate cement, freshly mixed, is very slightly antiseptic; old fillings have no action. Gutta-percha, as naturally suspected, is inactive.

These facts you will see clearly demonstrated on the plates which I now show you. You will also notice that I have strown some iodoform on one of the plates, and that the growth of bacteria has not thereby been interfered with in the least. It is a very remarkable phenomenon, which I am not able to explain, that certain preparations of gold also appear to be antiseptic. Pack's pellets, quarter-century gold foil, Abbey's foil, are all of this character, whereas other preparations which I have examined exhibit no antiseptic action. All preparations of gold lose their action on annealing. It has been supposed that this property of the gold is brought about by something added to it during the manufacture, or that the gold accumulates oxygen upon its surface, which, as we know, sometimes exerts an antibacterial action.

Another, perhaps more instructive, method of showing the antiseptic action of filling-materials is the following: Take a number of teeth extensively decayed, in which the pulp is, however, not exposed, excavate partially so as to leave a considerable quantity of carious dentine in each cavity; fill the cavities with the different materials to be tested, and put the teeth in a mixture of saliva and bread, where they should remain for some three days; then remove the teeth from the mixture, wash them and dry them and remove the fillings. The filling is most easily taken out by placing the crown of the tooth upon an anvil and giving it a short tap with a hammer; the filling usually flies out, exposing the untouched surface of dentine. Now remove a small particle of the carious dentine with a sterilized instrument, place it upon a sterile plate of nutritive agar-agar, then put the plate in a moist chamber at a temperature of about 38° C. In the course of two or three days the piece of dentine will be found to be surrounded by a whitish growth of fungi. If, however, the filling-material has exerted such an antiseptic action upon the dentine as to devitalize the fungi, or if the dentine has taken up a sufficient quantity of the antiseptic agent in the filling-material to become itself antiseptic, then no growth of fungi will form around the piece of dentine.

Having tested nearly all of the ordinary filling-materials by this method, I have found that pieces of dentine, taken from a tooth which had been filled with copper amalgam, never show any development of bacteria when brought upon the culture-plate, whereas pieces taken from teeth which had been filled with gold amalgam have invariably shown a fine growth of bacteria. The same is also the case with teeth filled with gutta-percha, with phosphate cement, and with tin-gold. In nearly all cases pieces of dentine from teeth filled with oxychloride of zinc have also given rise to a development of fungi, much less profuse, however, than that which forms around most other filling-materials.

From these results, it is very clear that copper amalgam exerts a very powerful antiseptic action upon the walls of the cavity containing it, and that there is no doubt that it thereby contributes greatly to prevent the recurrence of caries under the filling.

The idea that the combination of tin and gold is antiseptic is utterly fallacious, and the recommendation of this combination for filling root-canals, on the ground that it destroys micro-organisms by galvanic action, is unscientific and unfounded. It is a well-known fact that strong currents of electricity are required to exert any marked action upon the development of bacteria. We might, for instance, kill an ox by means of a shock of electricity, without doing any harm to the bacteria, which might be in him. I speak of this in particular because I know of more than one gentleman who has adopted the practice of filling root-canals with tin and gold, with this object in view.

DISCUSSION.

*Dr. Elliott.*—I would like to ask Dr. Miller whether these experiments do not go to prove that bacteria are the sole cause of the decomposition of the root-canals; generally speaking, we only consider it one of the causes; those who have had much to do with copper amalgam know that it does not stop decay.

*Dr. Miller.*—The antiseptic action of copper amalgam may diminish or cease altogether in the course of time. I only mean to say that, other things being equal, copper amalgam will prevent decay longer than the ordinary amalgams. There is no means by which a pulp can be decomposed except by bacteria. In caries the decomposition of the softened dentine is caused by bacteria; being an albuminoid substance, it is dissolved by the bacteria, just as an egg is dissolved in the stomach by the pepsin.

*Dr. Elliott.*—I think the most important factor is in the defect of the teeth, in construction, and has nothing to do with bacteria; otherwise, how could people who are not cleanly go through their lives without decay ?

*Dr. Jenkins.*—I should like to state that what Dr. Miller has arrived at, we, many of us, have also arrived at in a practical way. Most gentlemen have had very similar experience in regard to the untrustworthy nature of iodoform, and I am glad to hear Dr. Miller make this statement in regard to tin and gold; for he is one of those who thoroughly advocates the proper use of it. I believe those who have had the widest experience, claim for it only its advantages as a mechanical stopping. I should like to ask Dr. Miller if he has made use of the experiments which he has shown us, with oxychloride of zinc?

*Dr. Elliott.*—There is a general impression in the profession that the iodoform is decomposed.

*Dr. Mitchell.*—Mr. President, I don't know, but I must rather correct our president in regard to the decomposition of iodoform; that can be very easily established by using a hot-air syringe. The iodine can thus be liberated, and the reaction found there by the starch test. I have given my method of using it, placing a pledget of cotton saturated in alcohol and iodoform, and then throwing the hot air from the large bulb syringe through the cotton; this liberates free iodine.

*Dr. Elliott.*—Of course the only inference is, why not use iodine direct? The strongest tincture of iodine is the "Dental Tincture," recommended by Dr. Flagg, of Philadelphia.

*Dr. Miller.*—In answer to Dr. Elliott, let me say that we know by experience that caries does not take place on the exposed surfaces of the teeth, but we find caries appear in fissures and secluded spaces in teeth where the food lodges and where the fluids of the mouth do not circulate; consequently, the acids most active in caries cannot be those introduced into the mouth with foods, etc., but those formed within the mouth by fermentation.

Dr. Elliott speaks of the fissures in enamel and defects in developments of the teeth, etc.; these are what we call the *predisposing* causes of caries; they play a great part, and improperly developed teeth will become carious much sooner than teeth which are of good structure. We know very well, however, that cracks and fissures can never act as *exciting* causes of caries.

Dr. Elliott also mentioned that people who never cleaned their teeth are frequently exempt from decay. This, however, cannot be taken as proof that it is not advisable to keep the teeth properly clean, because in some cases the teeth are of such perfect structure that they do not become carious in spite of the uncleanliness. Moreover, it is a fact, especially with people who consume an excess of nitrogenous food, that, when the uncleanliness goes so far that putrefaction of the remains of food takes place, then we do not obtain an acid, but an alkaline reaction; and in this case, of course, we could not expect the occurrence of caries. It is for this reason that flesh-eating tribes—for instance, the Esquimaux—very rarely suffer from caries, because the food which they consume is such as does not give rise to acid reaction by fermentation. These flesh-eating tribes are less troubled with caries than the common house-dog.

In considering the subject of caries there are, of course, a vast number of different factors, predisposing as well as exciting, which must be taken into consideration, before we can come to a proper conclusion as regards the nature of dental caries. It would, however, lead us too far to enter into a discussion of this question at present. In regard to the question of Dr. Jenkins, I have tested oxychloride of zinc, and have found that in the fresh state it possesses considerable antiseptic action; it ceases to be antiseptic, however, soon after it has thoroughly hardened.

*Dr. Mitchell.*—I would like to ask Dr. Miller how he accounts for the preservation of teeth by gutta-percha?

*Dr. Miller.*—Gutta-percha fillings, if properly made, will exclude all particles of food from entering. Spaces do not form between the filling and the wall of the tooth where gutta-percha has been used, consequently fermentable substances cannot enter. We do not get the acid which must be there always before the dentine can be decalcified.

*Dr. Elliott.*—There is a peculiarity in regard to that. We know that an infinitesimal defect in a gold filling is fatal.

*Dr. Miller.*—If we have a small leakage in a gold filling, there is a tendency for the enamel to break away, and in the course of time the defect will get gradually greater. This tendency to crumble away does not exist around gutta-percha fillings; and herein lies their chief preservative power. The reason is not hard to find. Every time we bite on a large solid gold filling a shock or blow is thereby communicated to the walls of the cavity; and if these are not perfectly solid and intact, they will, in the course of time, suffer from these repeated blows. Gutta-percha fillings do not receive such blows, because they are not contoured, and the lighter blows which they do receive are not transmitted to the walls of the tooth.

*Dr. Mitchell.*—I should like to ask Dr. Miller how it is that, in removing a gutta-percha filling, provided the rubber dam has been applied, there is always a trace of moisture? That has been my experience with gutta-percha as a filling.

*Dr. Miller.*—I think I can make a gutta-percha filling which will seal the cavity thoroughly; besides, in case of an imperfect gold filling, you can insert an excavator between the filling and the walls of the cavity. This you cannot do in case of a gutta-percha filling.

*Dr. Mitchell.*—I cannot understand why this infinitesimal amount of moisture should not act in a detrimental manner to the teeth.

*Dr. Miller.*—I have an experiment which has been going on since 1882, which shows that saliva, when it putrefies or ferments, does not have any action upon the tooth structure; if your fluid, which appears between the filling of the teeth, can be renewed from time to time, and if the fluid contains sugar, which should ferment, then you would undoubtedly have, in the course of time, a detrimental action on the teeth. The very fact that it cannot be renewed would limit its action.

*Dr. D. Kelsey.*—I would like to ask the doctor, if it is meat which preserves the teeth of Esquimaux, what preserves the teeth of Hindoos, who never eat meat?

*Dr. Miller.*—Perhaps the healthy mode of life helps to prevent the caries; perhaps the physical character of the food; some kinds of food clean the teeth themselves. Wild races suffer much less from caries than we, because we have all our things cooked, and our teeth do not have the necessary gymnastic action. Finally, the chief answer that I have to give to the question is that the doctor is not quite right in his statement. Hindoo races are by no means exempt from caries, some of them suffering to a very great extent.

*Dr. Elliott.*—I can corroborate the statement. I find that in China there is a great deal of caries; not so much as in Europe; but were you to ask any foreigner if the Japanese have good teeth, he would say yes. I would like to add that, of all nations in the world,—and I have been in all countries,—the Japanese are far the worst in the matter of irregularities.

*Dr. Mitchell.*—Can our president give us any reason for this condition?

*Dr. Elliott.*—I could not say; at the time I went to Japan, there were a large number of dentists; they usually had their stands on the street, and, as a rule, they would make you a set of teeth in a very short time. It is to me a very remarkable thing that the Japanese get their knowledge of science from the Chinese, and have no artificial teeth whatever, except that they make two or three incisors merely for appearance. The Japanese are the inventors of the suction principle of plates. They are made of wood, very accurately, and the mastication, which is done on the back of the plate, which is covered with copper nails, is exceedingly useful. In regard to the amount of caries, we have no statistics to guide us in the matter. My student was the first Japanese who took up dentistry in Tokio, and he established a practice there.

*Dr. Jenkins.*—Is there not reason to believe that the Japanese are a mixed race, which may account for the irregularities?

*Dr. Elliott.*—I do not think that that is sufficient reason to account for it.

Subject passed.

(To be continued.)

